# Insights into the phylogeny of the ciliate of class Colpodea based on multigene data

**DOI:** 10.1002/ece3.9380

**Published:** 2022-10-12

**Authors:** Bailin Li, Yumeng Song, Tingting Hao, Li Wang, Weibin Zheng, Zhao lyu, Ying Chen, Xuming Pan

**Affiliations:** ^1^ Key Laboratory of Biodiversity of Aquatic Organisms Harbin Normal University Harbin Harbin P. R. China; ^2^ College of Life Sciences Northwest University Xi'an China; ^3^ School of Civil and Environmental Engineering Harbin Institute of Technology (Shenzhen) Shenzhen China

**Keywords:** ciliates, *Colpoda*, ITS1‐5.8S‐ ITS2 rRNA gene, mtSSU‐rRNA gene, phylogeny, SSU‐rRNA gene

## Abstract

In the class Colpodea, there are many unresolved evolutionary relationships among taxa. Here, we report 30 new sequences including SSU‐rRNA, ITS1‐5.8S‐ ITS2 rRNA, and the mitochondrial small subunit ribosomal RNA (mtSSU‐rRNA) genes of five colpodeans, and conduct phylogenetic analyses based on each individual gene and a two‐gene concatenated dataset. For the first time, multi‐genes were used to analyze phylogenetic relationships in the class Colpodea. The main findings are: (1) SSU‐rRNA, ITS1‐5.8S‐ ITS2 rRNA, and mtSSU‐rRNA gene sequences of *C*. *reniformis* and *C*. *grandis* are provided for the first time, and these two species group into the clade including *C*. *inflata*, *C*. *lucida*, *C*. *cucullus*, and *C*. *henneguyi*; (2) clustering pattern and morphological similarity indicate that *Bresslauides discoideus* has a close relation with Colpodidae spp.; (3) *Emarginatophrya* genus diagnosis is improved to be ‘Hausmanniellidae with sharply shortened and isometric leftmost 1‐4 ciliary rows’ and *Colpoda elliotti* is transferred to *Emarginatophrya*; (4) the genus *Colpoda* is still non‐monophyletic with the addition of 10 populations from five *Colpoda* species sequences, but there are only two *Colpoda* groups left based on the present work: Group I comprises *C*. *inflata*, *C*. *lucida*, *C*. *cucullus*, *C*. *henneguyi*, *C*. *reniformis*, and *C*. *grandis*, Group II comprises *C*. *maupasi* and *C*. *ecaudata*, and the presence of diagonal grooves and the way the vestibular opens might be the two key features that differentiates *Colpoda* species groups; (5) a close molecular relationship, and highly similar merotelokinetal mode, somatic ciliary pattern, and basic organization of the oral apparatus with *P*. *steinii* suggests *Bromeliothrix metopoides* should be temporarily assigned to Colpodidae.

## Graphical abstract

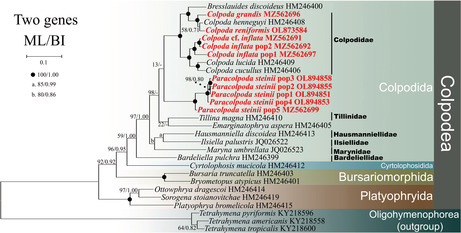

## 1 INTRODUCTION

Ciliates are a highly differentiated group of microbial eukaryotes that inhabit virtually all environments on the Earth's surface where there is sufficient water for their survival. They play a fundamental role in microbial food web function by mediating the transfer of organic matter and energy between different trophic levels in a wide range of ecosystems (Agatha et al., 2021; Bai et al., 2020; Chi et al., 2021; Dias et al., 2021; Liu et al., 2022; Song et al., 2021; Wang et al., 2021; Weisse & Montagnes, 2021; Wu et al., 2021). Due to their particular nature (e.g., high reproductive capacity, diverse morphology, nuclear dimorphism, chromosomal fragmentation, and sensitivity to environmental changes), ciliates are important model organisms in many disciplines, such as epigenetics, symbiotic relationships, molecular biology, organismal development, evolution, biogeography, and ecology (Berger, 2011; Cheng et al., 2019; Corliss, 1979; Gao et al., 2014; Lynn, 2008; Song et al., 2009; Zhao et al., 2020). Despite this attention, however, there are numerous unresolved issues concerning ciliate phylogeny and systematics (Corliss, 1979; Gao et al., 2016).

Ciliates of the class Colpodea (Small & Lynn, 1981) are an important structural component of soil protozoa. They have been reported from terrestrial habitats world‐wide from the driest deserts to permanently saturated wetlands and bogs (Bourland et al., 2012; Foissner, 1993; Foissner et al., 2014; Vďačný & Foissner, 2019). Characteristically, colpodeans produce resting cysts that allow them to survive desiccation and other adverse environmental conditions and thus often constitute a significant hidden soil biodiversity component (Foissner, 1987; Quintela‐Alonso et al., 2011). Although their oral apparatus is highly diverse, colpodeans share a similar somatic cortex structure which has led to misclassification among species (Foissner, 1993; Lynn, 2008; Quintela‐Alonso et al., 2011; Vďačný & Foissner, 2019). Small and Lynn (1981) recognized colpodeans as a monophyletic taxon and established the class Colpodea on the basis of a set of unique morphological characteristics. The application of molecular techniques supported Colpodea monophyly and its position within the subphylum Intramacronucleata (Dunthorn et al., 2014; Lynn, 2003), but also resulted in a major re‐evaluation of interclass classification and evolution (Foissner et al., 2011; Vďačný & Foissner, 2019).

Four major Colpodea lineages (Bursariomorphida, Platyophryida, Cyrtolophosidida, and Colpodida) have been identified by phylogenetic inference based on nuclear small subunit ribosomal RNA (nSSU‐rRNA) sequences (Dunthorn et al., 2008; Foissner et al., 2011; Vďačný & Foissner, 2019). Among these, Bursariomorphida (Fernández‐Galiano, 1979) was found to include in the taxa previously assigned to the order Bryometopida (Foissner, 1985; Vďačný & Foissner, 2019); the position of Platyophryida is indeterminate (Bourland et al., 2011; Rajter et al., 2020), Cyrtolophosidida is divided into two main clades, and Colpodida is the most complex clade (Foissner et al., 2011). Dunthorn et al. (2014) investigated Colpodea phylogeny based on mtSSU‐rRNA gene sequence data and reported the same four lineages as those from the nSSU‐rRNA analyses mentioned earlier. Nevertheless, there are inconsistencies in the evolutionary relationships among taxa within Colpodea (e.g., the position of Platyophryida), indicating that phylogenetic analysis based on a single gene is problematic (Rajter et al., 2020). Furthermore, species sampling remains unbalanced in Clopodea (e.g., there is only one species in Ilsiellidae and Bardeliellidae) that has led to the formation of paraphyletic groups and uncertainty in the assignment of various taxa (Fan et al., 2013; Foissner & Stoeck, 2009; Quintela‐Alonso et al., 2011; Strüder‐Kypke et al., 2000; Vďačný & Foissner, 2019; Zhao et al., 2016).

Recently, investigations based on multigene trees have been increasingly used for phylogenetic studies and generally produce more robust results than those based on single gene markers (Gao et al., 2013, 2016, 2017; Sun et al., 2020; Wang, Wang, et al., 2017; Zhang et al., 2020). Multigene analyses usually include nSSU‐rRNA, ITS1‐5.8S‐ITS2 and nLSU‐rRNA genes, which are in the same chromosome (Wang, Wang, et al., 2017). However, Colpodea phylogenetic analyses so far have focused on a single gene, (i.e., the nSSU‐rRNA gene or the mtSSU‐rRNA gene), and few studies have used a multigene approach (e.g., Dunthorn et al., 2011). Furthermore, most colpodean sequences in the NCBI database are of SSU‐rRNA, while other gene sequences are very limited. This is one of the main dilemmas of colpodean phylogenetics.

With the conservative evolution of SSU rDNA alongside various issues such as asynchronous evolution with morphology, delineation of Colpodea species remains problematic. And, the usefulness of combination of nuclear and mitochondrial gene is rarely tested in Colpodea. The mtSSU‐rRNA gene is more variable and has a higher evolutionary rate compared to the nSSU‐rRNA gene (Boore & Brown, 1998; Moore, 1995; Wang, Zhang, et al., 2017), and it is reasonable to assume that it can effectively uncover deep nodes within Colpodea (Dunthorn et al., 2011, 2014; Rand, 2003; Zhang et al., 2019). In this study, 30 new sequences of 10 populations from five colopdeans (*Colpoda* cf. *inflata*, *C. reniformis*, *C. inflata* pop1‐2, *C. grandis*, and *Paracolpoda steinii* pop1‐5) isolated from soil and water samples in Harbin, Shuangyashan and Mudanjiang, China, are presented. For the first time, Colpodea phylogenetic analyses based on multiple genes (SSU‐rRNA gene, mtSSU‐rRNA gene), along with analyses of morphological data are carried out.

## 2 MATERIALS AND METHODS

### 2.1 Taxon sampling, observation, and terminology

Soil and water samples of nine populations of four colopodeans were obtained from several sites in Harbin, Shuangyashan and Mudanjiang, Heilongjiang province, northern China. *Colpoda* cf. *inflata*, *C. inflata* pop1 (population 1), and *Colpoda grandis* were collected from a farmland at the Hulan Beet Research Institute of Heilongjiang University (45° 59′ 47″ N, 126° 38′ 18″ E), Harbin. *Colpoda inflata* pop2 was collected from an experimental plot in Harbin Normal University (45° 86′ 46″ N, 126° 56′ 15″ E), Harbin. *Colpoda reniformis* was collected from Mudanjiang Xuanwu Lake in National Agricultural Park (44° 9′ 10″ N, 129° 9′ 19″ E), Mudanjiang. *Paracolpoda steinii* pop1‐3 were collected from several waters in Qixing River wetland (46° 41′ 20″ N, 132° 2′ 30″ E; 46° 42′ 48″ N, 132° 11′ 36″ E; 46° 43′ 41″ N, 132° 18′ 12″ E), Shuangyashan, and *Paracolpoda steinii* pop4‐5 were collected from soil at the same location (46° 42′ 59″ N, 132° 8′ 21″ E; 46° 42 13″ N, 132° 7′ 29″ E). Ciliates were isolated from soil using the non‐flooded Petri dish method at room temperature (ca. 25°C) with rice or wheat grains added to enrich bacteria growth as a food source. Clonal cultures were established for each of the 10 populations from five colpodean species isolated. Silver carbonate staining (Foissner, 1992) was used to reveal the infraciliature. Five slides containing silver carbonate‐stained voucher specimens encircled in black ink are deposited in the Laboratory of Protozoology, Harbin Normal University of China with registration numbers HTT‐20211006‐01; −20,211,006‐02; −20,211,006‐03; −20,211,006‐04 and −2,021,100,605. Terminology and classification was mainly according to Foissner et al. (2011) and Lynn (2008).

### 2.2 DNA extraction, amplification, and sequencing

For each species, 1–10 cells from clonal culture were isolated under a stereomicroscope using a micropipette, washed with distilled water at least thrice to remove potential contaminants, and then incubated in non‐nutrient distilled water for 24 h. Cells were then transferred to an Eppendorf tube in a volume of no more than 5 μl distilled water. Total genomic DNA was extracted using the DNeasy & Tissue Kit (Hilden, QIAGEN) following the manufacturer's instructions.

SSU‐rRNA, ITS1‐5.8S‐ITS2 rRNA, and mtSSU‐rRNA genes were amplified by the polymerase chain reaction (primers see Appendix Table A1). High‐fidelity Taq polymerase (Takara Ex Taq; Takara Biomedicals) was used to reduce amplification errors. PCR condition for the SSU‐rRNA gene amplification was denaturation for 5 min at 94°C, followed by 5 cycles of denaturation for 30 s at 94°C, annealing for 1 min 45 s at 56°C, extension for 2 min at 72°C and the other 25 cycles of denaturation for 45 s at 94°C, annealing for 1 min 45 s at 60°C, extension for 2 min at 72°C and a final extension at 72°C for 8 min. The ITS1‐5.8S‐ITS2 rRNA gene was amplified as follows: 5 min initial denaturation 94°C, followed by 35 cycles of denaturation for 30 s at 94°C, annealing for 45 s at 58°C, extension for 1 min at 72°C and a final extension at 72°C for 10 min. The mtSSU‐rRNA gene was amplified as follows: 5 min initial denaturation 94°C, 5 cycles of 45 s at 94°C, 1 min 45 s at 58°C and 2 min at 72°C, and the other 25 cycles of denaturation for 45 s at 94°C, annealing for 1 min 45 s at 60°C, and extension for 2 min at 72°C with a final extension of 10 min at 72°C. Cloning and sequencing were both routine operations (Zhang et al., 2019). A total of 30 new SSU‐rRNA, ITS1‐5.8S‐ ITS2 rRNA, and mtSSU‐rRNA gene sequences from five Colpodea species were determined (GenBank accession numbers see Appendix Table A2).

### 2.3 Data sets and alignments

**FIGURE 1:**
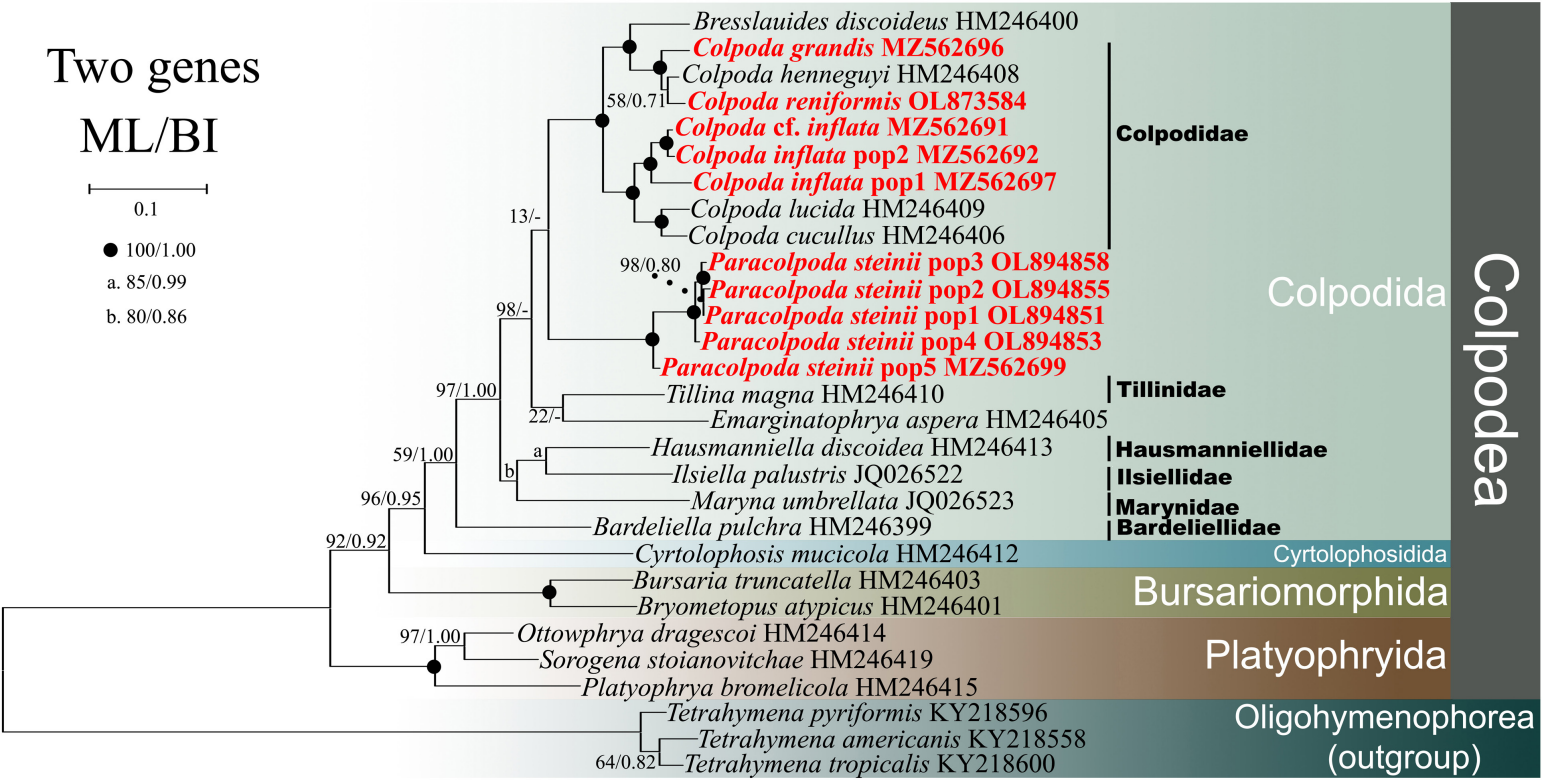
The maximum‐likelihood (ML) tree based on the concatenated genes (SSU‐rRNA and mtSSU‐rRNA genes) of major members of the class Colpodea. Newly added sequences in this study are bolded in red. Node support is shown as: ML bootstraps/BI posterior probability. “‐” indicate mismatch in topology between Bayesian and ML tree. Fully supported (100%/1.00) branches are marked with solid circles. The scale bar corresponds to five substitutions per 100 nucleotide sites.

**FIGURE 2:**
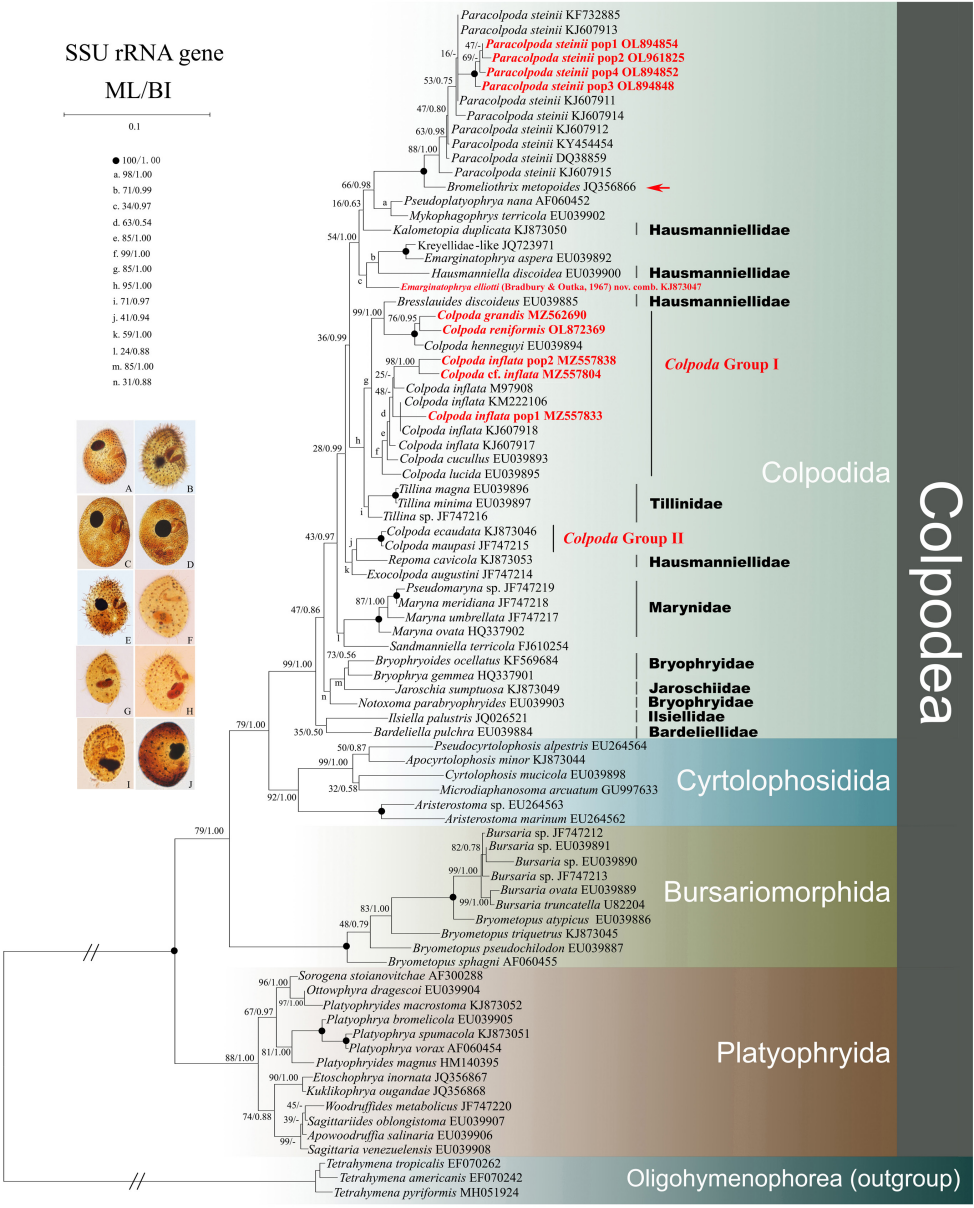
The maximum‐likelihood (ML) tree based on the SSU‐rRNA gene of major members of the class Colpodea. Newly added sequences in this study are bolded in red type. Node support is shown as: ML bootstraps/BI posterior probability. “‐” indicate mismatch in topology between Bayesian and ML tree. Fully supported (100%/1.00) branches are marked with solid circles. Two long branches has been shortened, as shown by “//”, and the other branches are drawn to scale. The scale bar corresponds to five substitutions per 100 nucleotide sites. (a–j) The five newly sequenced species after silver carbonate staining. (a, b) *Colpoda inflata* pop1‐2; (c) *Colpoda grandis*; (d) *Colpoda reniformis*; (e) *Colpoda* cf. *inflata*; (f–j) *Paracolpoda steinii* pop1‐5.

**FIGURE 3:**
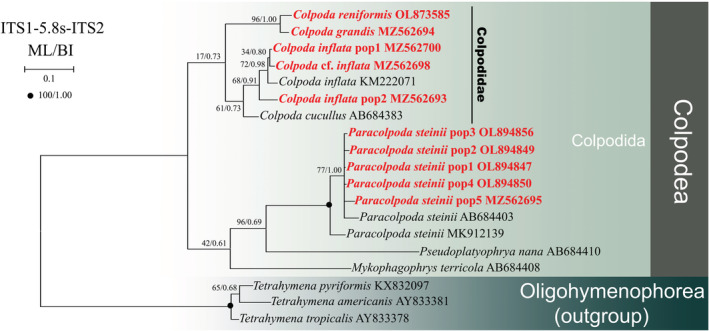
The Bayesian inference (BI) tree based on the ITS1‐5.8S‐ITS2 gene of major members of the class Colpodea. Newly added sequences in this study are bolded in red type. Node support is shown as: ML bootstraps/BI posterior probability. “‐” indicate mismatch in topology between Bayesian and ML tree. Fully supported (100%/1.00) branches are marked with solid circles. The scale bar corresponds to 0.2 expected substitutions per site.

**FIGURE 4:**
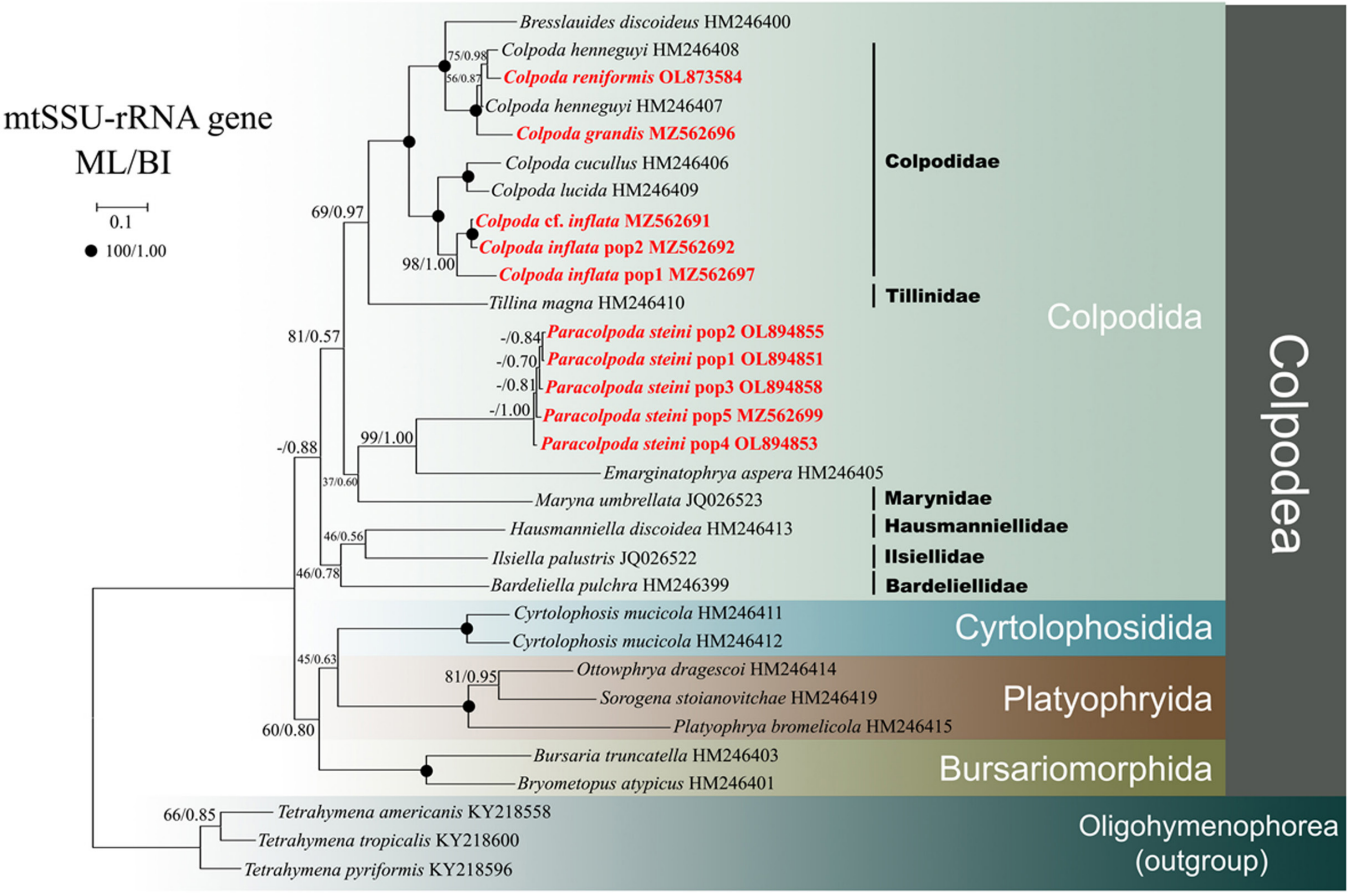
The Bayesian inference (BI) tree based on the mtSSU‐rRNA gene of major members of the class Colpodea. Newly added sequences in this study are bolded in red type. Node support is shown as: ML bootstraps/BI posterior probability. “‐” indicate mismatch in topology between Bayesian and ML tree. Fully supported (100%/1.00) branches are marked with solid circles. The scale bar corresponds to 0.1 expected substitutions per site.

Other colpodeans and outgroup sequences were obtained from GenBank, and their accession numbers are shown in Figures 1, 2, 3, 4. Sequences downloaded with questionable morphological information, unknown source, or of too short length were not included. SSU‐rRNA gene was aligned using Clustal W implemented in BioEdit 7.0.1 (Hall, 1999). The ITS1‐5.8S‐ITS2 rRNA gene and mtSSU‐rRNA gene sequences were aligned using the default parameters implemented in Guidance server (http://guidance.tau.ac.il/) (Penn et al., 2010). To remove ambiguously aligned positions in the multiple sequence alignments all the sequence datasets were edited by eye in BioEdit 7.0.1 (Hall, 1999). Sequences used for phylogenetic analyses were compiled into four data sets: (i) 1833 characters of SSU‐rRNA (83 taxa); (ii) 722 characters of ITS1‐5.8S‐ rRNA –ITS2 (19 taxa); (iii) 1138 characters of mtSSU‐rRNA (31 taxa); and (iv) 2895 characters of concatenated sequence data of the above two genes (29 taxa in total). The two alignments were concatenated to be contiguous in SeaView V4 for gene analysis. All new sequences were deposited in the NCBI database (https://www.ncbi.nlm.nih.gov/), accession numbers, lengths, and G&C contents are shown in Appendix Table A2. For all trees, the outgroup was composed of three species of tetrahymenid taxa (*Tetrahymena pyriformis*, *T*. *tropicalis* and *T*. *americanis*).

### 2.4 Phylogenetic analyses

Phylogenetic trees were inferred using maximum likelihood (ML) and Bayesian inference (BI) methods. ML analyses were constructed by RAxML‐HPC2 v8.2.12 (Stamatakis, 2014), and BI analyses by MrBayes v3.2.7a (Ronquist et al., 2012), both on the CIPRES Science Gateway (URL: http://www.phylo.org/sub_sections/portal). The most appropriate model for phylogenetic analyses of nSSU‐rRNA gene (SSU‐rRNA, ITS1‐5.8S‐ITS2 rRNA gene) was GTR + I + G as selected by Modeltest v3.4 (Posada & Crandall, 1998). The ML and BI trees based on mtSSU‐rRNA gene and concatenated dataset were constructed according to the GTR + I + G model chosen by MrModeltest v.2.2 program (Nylander, 2004). ML analysis was conducted using rapid bootstrap with 1000 non‐parametric bootstrap replicates. Bayesian posterior probabilities were calculated by running four chains for 10,000,000 generations, with cold chain sampling every 10,000 generations. The first 25% of sampled trees were discarded as burn‐in. Support value <70%/0.94 (ML/BI) was considered as low, 70%–94% (ML) as moderate, and >95%/0.95 (ML/BI) as high. MEGA 7.0 (Kumar et al., 2016) was utilized to visualize tree topologies.

### 2.5 Sequence analyses and putative secondary structure modeling

The secondary structures of ITS2 sequences of *Paracolpoda steinii* pop1‐5, *Colpoda* cf. *inflata* and *C*. *inflata* pop2 were predicted based on the models of *Cardiostomatella vermiformis* (EU262621) (http://www.rna.ccbb.utexas.edu) using the Mfold website (http://unafold.rna.albany.edu/?q=mfold/RNA‐Folding‐Form) with default settings (Miao et al., 2008; Zuker, 2003). The 5′ and 3′ ends of the ITS2 sequences were determined via Rfam (available on the web http://www. sanger.ac.uk/Software/Rfam/; Griffiths‐Jones et al., 2003).

## 3 RESULTS

### 3.1 Phylogeny based on concatenated dataset

The phylogenetic trees constructed using ML and BI had similar topologies, therefore, only the ML trees and their support values from both methods are shown in Figure 1. The order Colpodida contains six families (Colpodidae, Tillinidae, Hausmanniellidae, Ilsiellidae, Marynidae, and Bardeliellidae; Figure 1). However, due to sequence paucity, there is only one available sequence from single species in the Tillinidae, Hausmanniellidae, Ilsiellidae, Marynidae, and Bardeliellidae. *Hausmanniella discoidea* and *Ilsiella palustris* form a sister clade (85% ML, 0.99 BI). One Marynidae species (*Maryna umbrellata*), *H*. *discoidea* and *I*. *palustris* cluster together to form a clade (80% ML, 0.86 BI). The family Tillinidae is represented by one species (*Tillina magna*) and *Emarginatophrya aspera* cluster together to form a sister clade. The family Bardeliellidae (*Bardeliella pulchra*) is the sister of the order Colpodida. The order Cyrtolophosidida and Colpodida cluster together with high support (96% ML, 0.95 BI). The class Colpodea is monophyletic with four main lineages (Platyophryida, Bursariomorphida, Cyrtolophosidida, and Colpodida), each of which is monophyletic. Platyophryida is the deepest branching lineage within the Colpodea. Bursariomorphida is a sister group to the clade formed by the orders Colpodida and Cyrtolophosidida.

The newly sequenced species *C*. *reniformis* and *C*. *henneguyi* cluster as a sister group, and the resulting clade then groups with the other newly sequenced species *C*. *grandis* (100% ML, 1.00 BI) and *Bresslauides discoideus* (100% ML, 1.00 BI). The two newly sequenced *Colpoda* cf. *inflata* and *C*. *inflata* pop2 cluster together with full support (100% ML, 1.00 BI), and the resulting clade then groups with the clade formed by newly sequenced *C*. *inflata* pop1 (100% ML, 1.00 BI). The genus *Colpoda* is monophyletic.

The five newly sequenced populations of *Paracolpoda steinii* (1–5) cluster together. The five new populations group cluster in the Colpodida clade ‘core position’. Noticeably, *Paracolpoda steinii* pop1 is a sister to *P*. *steinii* pop2 (98% ML, 0.80 BI) and forms a sister group with *P*. *steinii* pop3 (100% ML, 1.00 BI) and *P*. *steinii* pop4 (100% ML, 1.00 BI). *P*. *steinii* pop5 is a sister to this clade (100% ML, 1.00 BI).

### 3.2 Phylogenetic analyses based on SSU‐rRNA gene sequence data

Each of the Colpodea four orders are monophyletic and the genus *Colpoda* is non‐monophyletic. The orders Colpodida and Cyrtolophosidida cluster together to form a clade that is a sister to Bursariomorphida. The order Platyophryida is the deepest branching lineage within the Colpodea.

The order Colpodida contains eight families (Colpodidae, Tillinidae, Grossglockneriidae, Bryophryidae, Hausmanniellidae, Ilsiellidae, Marynidae, and Bardeliellidae; Figure 2). *Hausmanniella discoidea* clusters with Kreyellidae‐like taxa and *Emarginatophrya aspera* (71% ML, 0.99 BI). The family Grossglockneriidae is represented by two species (*Mykophagophrys terricola* and *Pseudoplatyophrya nana*) that cluster together with high support (98% ML, 1.00 BI). Family Bryophryidae is monophyletic. *Jaroschia sumptuosa*, *Bryophrya gemmea*, and *Bryophryoides ocellatus* cluster together as a clade that then forms a sister clade with *Notoxoma parabryophryides*. *Bardeliella pulchra* and *Ilsiella palustris* cluster together to form a clade. Family Tillinidae is monophyletic and contains three species. Marynidae is paraphyletic because *Maryna meridiana* and *Pseudomaryna* sp. cluster together with full support and then group with *M. umbrellata* (87% ML, 1.00 BI). *Maryna ovata* clusters with the *M*. *meridiana* + *Pseudomaryna* sp. + *M*. *umbrellata* clade with a full support. Marynidae has a sister relationship with *Sandmanniella terricola*.

Two Colpodidae species (*C*. *ecaudata* and *C*. *maupasi* JF747215) and *Repoma cavicola* cluster together to form a clade that is a sister group to *Exocolpoda augustini*. *Colpoda ecaudata* and *C*. *maupasi* (JF747215) cluster together with full support but far away from most congeners. *Colpoda elliotti* is a sister to the clade formed by *Hausmanniella discoidea*, Kreyellidae‐like taxa and an *Emarginatophrya aspera* cluster. The five newly sequenced species cluster at the Colpodida clade core position. *Colpoda reniformis* and *C*. *grandis* are sisters with moderate support (76% ML, 0.95 BI) as a sister group to *C*. *henneguyi* with full support. The clade formed by *C*. *inflata* pop2 and *Colpoda* cf. *inflata* (98% ML, 1.00 BI) is a sister to another *C*. *inflata* population (M97908), while *C*. *inflata* pop1 clusters with the other two *C*. *inflata* populations (KM222106 and KJ607918). The 12 *Paracolpoda steinii* sequences including four newly sequenced species (*P*. *steinii* pop1‐4) cluster together with *Bromeliothrix metopoides*. In the SSU‐rRNA gene tree (Figure 2), *Bardeliella pulchra* clustered with *Ilsiella palustris*.

### 3.3 Phylogenetic analyses based on ITS1‐5.8S‐ITS2 region sequence data and the secondary structures of ITS2

In the ITS1‐5.8S‐ITS2 rRNA gene tree, bootstrap values for the maximum likelihood (ML) and posterior probabilities for the Bayesian inference (BI) were mapped onto the best BI tree (Figure 3). Due to sequence shortages, only the order Colpodida (family Colpodidae, genus *Colpoda* and *Paracolpoda*) and outgroup were included in this tree. Colpodida, *Colpoda*, and *Paracolpoda* form a monophyletic clade. *Colpoda reniformis* is still separated from the other newly sequenced *Colpoda* species (*C*. *inflata* pop1‐2 and *Colpoda* cf. *inflata*), and forms a sister clade with the newly sequenced *C*. *grandis* (96% ML, 1.00 BI). Two populations of *C*. *inflata* (KM222071 and MZ562700), *C*. *inflata* pop1, and *Colpoda* cf. *inflata* cluster together, and the clade then forms a sister clade with *C*. *inflata*. *Colpoda inflata* pop2 cluster with the *C*. *inflata + C*. *inflata* pop1 + *Colpoda* cf. *inflata* clade. Seven *Paracolpoda* species cluster in one clade, and the clade formed by the five newly sequenced *Paracolpoda steinii* cluster as a sister group to *P*. *steinii* (AB684403) with moderate support (77% ML, 1.00 BI). *Paracolpoda steinii* (AB684403) groups with the *P*. *steinii* pop1‐5 clade, which then clusters with *Paracolpoda steinii* MK912139 with full support.

**FIGURE 5:**
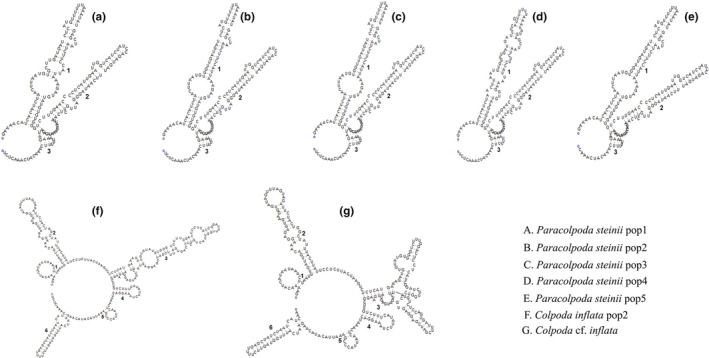
The putative secondary structures of ITS2 in the present study.

Secondary structures of the ITS2 transcript of the seven species are shown in Figure 5. The same taxa shared a very similar pattern of secondary structure. Only three helice spins were found in the genus *Paracolpoda* while six in *Colpoda*. There are differences in the helix 1 among different populations of the genus *Paracolpoda*, while mainly in helix 3 in the genus *Colpoda*.

### 3.4 Phylogenetic analyses based on mtSSU‐rRNA gene sequence data

The BI tree topology is similar to the ML tree, so only the BI tree with branch‐support values is shown in Figure 4. The mtSSU‐rRNA and SSU‐rRNA gene phylogenies show similar relationships within Colpodea. Noticeably, both the order Colpodida and the family Colpodidae are monophyletic. *Bardeliella pulchra* groups within the Colpodida clade.

Within Colpodea, the order Bursariomorphida is a sister to the clade formed by Platyophryida and Cyrtolophosidida. The newly sequenced species *C*. *reniformis* and *C*. *henneguyi* HM246408 cluster with another population of *C*. *henneguyi* HM246407, and the clade then clusters with *C*. *grandis* (ML 100%, BI 1.00). The results shown above are similar with those of concatenated and SSU‐rRNA gene phylogenies. The five new *Paracolpoda steinii* populations cluster together and form a sister clade with *Emarginatophrya aspera* (ML 99%, BI 1.00). Three *Colpoda* species (*C*. *inflata* pop1‐2 and *Colpoda* cf. *inflata*) cluster in one group, *C*. *inflata* pop2 and *Colpoda* cf. *inflata* form a sister clade with a full support, and the resulting clade then forms a sister relationship with *C*. *inflata* pop1 (ML 98%, BI 1.00). The clustering pattern of the three *Colpoda* species is relatively stable in all phylogenetic trees. Due to sequence shortages, there are only single species sequences available for Tillinidae, Ilsiellidae, Marynidae, and Bardeliellidae. *Tillina magna* clustered with Colpodida, *Maryna umbrellata* is a sister to the clade formed by five new *Paracolpoda steinii* populations and *Emarginatophrya aspera*.

## 4 DISCUSSION

### 4.1 Phylogeny of genus *Colpoda*

In previously reported SSU rRNA gene trees, typical *Colpoda* species are distributed over the whole Colpodea tree, and mainly form four groups: *C*. *inflata* + *C*. *cucullus* + *C*. *lucida*, *C*. *henneguyi*, *C*. *maupasi* + *C*. *ecaudata*, and *C*. *elliotti* clades (Foissner et al., 2011, 2014; Foissner & Stoeck, 2009; Lynn et al., 1999; Vďačný & Foissner, 2019). This motivated Foissner et al. (2011) and Dunthorn et al. (2012) to suggest rapid *Colpoda* radiation that produced several new genera and families. We added 30 new multiple gene sequences of 10 populations from five colopdeans. These additions change the *Colpoda* species into three main groups: Group I is composed of *C*. *inflata*, two Chinese *C*. *inflata* populations, *Colpoda* cf. *inflata*, *C*. *lucida*, *C*. *cucullus*, *C*. *henneguyi*, *C*. *reniformis*, and *C*. *grandis*; Group II comprises *C*. *maupasi* and *C*. *ecaudata*; and Group III only contains *C*. *elliotti* in the SSU rRNA gene trees.

Notably, for the first time, we provide *C*. *reniformis* and *C*. *grandis* SSU‐rRNA, ITS1‐5.8S‐ ITS2 rRNA, and mtSSU‐rRNA gene sequences. *Colpoda reniformis* and *C*. *grandis* clustered with *C*. *henneguyi*, and the resulting clade then clustered with *Bresslauides discoideus*. In this case, *B*. *discoideus* groups into the *Colpoda* clade including *C*. *inflata*, *C*. *lucida*, *C*. *cucullus*, *C*. *henneguyi*, *C*. *reniformis*, and *C*. *grandis* in the SSU rRNA, mtSSU‐rRNA gene, and concatenated trees. *Bresslauides* species differ mainly by the unique right semicircular oral polykinetid that is longer than the left, as opposed to being of equal length in the Colpodidae (Foissner, 1987, 1993), but similar with the features of Colpodidae sp. in vivo, which indicates that *Bresslauides* species have a close relation with Colpodidae sp. Broader sampling and molecular sequencing of *Bresslauides* species would be necessary to clarify this relationship, although we also believe that the character of the right semicircular curved oral polykinetid may have evolved more than once (Bourland et al., 2011; Dunthorn et al., 2008).

*Colpoda elliotti* KJ873047 clusters in the clade formed by Kreyellidae‐like species, *Emarginatophrya aspera*, and it unites with *E*. *aspera* in trees comprised colpodids only (Foissner et al., 2011). *Emarginatophrya*, morphologically distinguished by a distally emarginated left oral polykinetid, was established by Foissner (2016). However, establishing this new colpodid genus has not helped to completely erase the *Colpoda* paraphyly problem. *Colpoda aspera* and *C*. *elliotti* are highly similar morphologically and according to Foissner (1993), ‘the left oral polykinetid of *C*. *elliotti* is slightly variform’. Then, we found in fig. 53b in Foissner's book that the left oral polykinetid was slightly emarginated (as a result of its leftmost kinety always consisting of only two basal bodies) in some representative individuals. The only two *Emarginatophrya* species, *E*. *aspera* and *E*. *terricola*, also have three to four sharply shortened leftmost ciliary rows (almost of equal length), which were described as ‘emarginated left oral polykinetid’. Therefore, we decided to change the diagnosis of *Emarginatophrya* to *‘*Hausmanniellidae with sharply shortened and isometric leftmost 1–4 ciliary rows*’*. So, we transferred *Colpoda elliotti* to *Emarginatophrya*, and establish a new combination *Emarginatophrya elliotti* (Bradbury & Outka, 1967) nov. comb., which partially solves the *Colpoda* problem.

As a result, there are now two remaining *Colpoda* groups: Group I comprises *C*. *inflata*, *C*. *lucida*, *C*. *cucullus*, *C*. *henneguyi*, *C*. *reniformis*, and *C*. *grandis*, and Group II comprises *C*. *maupasi* and *C*. *ecaudata*. *Colpoda maupasi* and *C*. *ecaudata* group with *Exocolpoda* and *Ropoma* species, which have distinctly different characteristics from the former species, including different life cycles, boomerang‐shaped left oral ciliary fields, and a very thick resting cyst wall. *Colpoda maupasi* and *C*. *ecaudata* are two common *Colpoda* species, but they group far away from other congeners. A probable explanation for this grouping pattern is that both *C*. *maupasi* and *C*. *ecaudata* lack the diagonal groove and their vestibular opening is marked on the left body margin by shallow indentation, but other *Colpoda* species having molecular information possess diagonal grooves (except *C*. *inflata*, but its left body margin with right‐angular notch, in the center where the vestibulum opens; Foissner, 1993). Morphologically, *Tillina magna* also possess diagonal grooves (Foissner, 1993), which might explain why *Tillina magna* and the Group I of the genus *Colpoda* cluster together in the SSU‐rRNA and mtSSU‐rRNA gene trees. Collectively, the presence of diagonal grooves and the way the vestibular opens might be the two key features that differentiate *Colpoda* species groups. Due to sequence shortages, only one species of Tillinidae used in the concatenated tree, which leads to the clustering of *Tillina magna* with *Emarginatophrya aspera*. Therefore, we suggest that sampling should be expanded to further address this issue.

### 4.2 *Paracolpoda* and *Bromeliothrix* phylogeny

Totally twelve populations (including five from the present work) of *Paracolpoda steinii* firmly cluster with *Bromeliothrix metopoides* in the SSU rRNA gene trees. *Bromeliothrix metopoides* has the same merotelokinetal mode with *Paracolpoda steinii*, and its somatic ciliary pattern is similar to that of *Colpoda* species (Foissner, 1993, 2010; Weisse et al., 2013). *Bromeliothrix* has a ciliary and silverline pattern typical for members of the family Colpodidae, and its basic organization of the oral apparatus is also the same as that known from genera of Colpodidae families (Foissner, 2010; Foissner et al., 2002). Foissner pointed out that *Bromeliothrix* neither belong to the Exocolpodidae nor the Hausmanniellidae due to its complex morphological characteristics, and several phylogenetic analyses based on SSU rRNA gene sequence also indicated that *B*. *discoideus* was more closely related to a clade formed by large *Colpoda* species than with *Hausmanniella discoidea*, a type of the hausmanniellids (Bourland et al., 2011; Dunthorn et al., 2008). The *Paracolpoda* genus was assigned to the Colpodidae and established by Lynn (1978), who noted that *Colpoda* species have a somatic groove while *Paracolpoda* species do not. Foissner (1985) subsumed *C*. *steini* into *Paracolpoda*. A close molecular relationship, highly similar merotelokinetal mode, somatic ciliary pattern and basic organization of the oral apparatus with *P*. *steinii* suggests *Bromeliothrix metopoides* could be temporarily assigned to Colpodidae (Foissner, 1993, 2010, Foissner et al., 2002, 2011). Additionally, the rather long, semicircular curved right oral polykinetid character, among other features of the Hausmanniellidae (Foissner, 1993) might be interpreted as convergence. Until now, the molecular data available in the NCBI database for *Bromeliothrix* and *Paracolpoda* are very sparse, with sequences from only one species for each. Broader sampling and molecular sequencing of *Paracolpoda* species is necessary to further clarify these relationships. *Paracolpoda steinii* pop5 separates from *P. steinii* pop1‐4 in concatenated tree, the reason may be due to morphological differences, e.g., a larger body size compared with the other four populations.

### 4.3 *Bardeliella pulchra* evolutionary position

In previous work, and in our study, *B*. *pulchra* was always the earliest divergent branch of Colpodida in the SSU‐rRNA gene tree, which means it has been stable between Colpodida and Cyrtolophosidida (Bourland et al., 2011; Dunthorn et al., 2012; Rajter et al., 2020; Vďačný & Foissner, 2019). But, we established multiple gene trees and they all confirmed the instability of the position of *B*. *pulchra* in Colpodida.

Morphologically, three traits indicated that *Bardeliella pulchra* belongs to the Colpodida: (1) like most of colpodids, *B*. *pulchra* can divide in cysts; (2) *B*. *pulchra*'s silverline pattern consists of highly ordered, comparatively large meshes extending between two ciliary rows each, and this silverline pattern is widely found in Colpodida species (Foissner, 1993; Foissner et al., 2011); (3) the oral ciliary fields of *B*. *pulchra. Bardeliella* and *Colpoda* are similar, i.e., the left oral ciliary field consisting of a shorter proximal portion with equidistantly spaced, monokinetidal rows (Foissner et al., 2011).

## AUTHOR CONTRIBUTIONS

**Bailin Li:** Formal analysis (lead); software (lead); writing – original draft (lead); writing – review and editing (equal). **Yumeng Song:** Formal analysis (equal); investigation (equal); software (equal). **Tingting Hao:** Formal analysis (equal); investigation (equal); software (equal). **Li Wang:** Formal analysis (equal); investigation (equal); software (equal). **Weibin Zheng:** Formal analysis (equal); investigation (equal); software (equal). **Zhao Lyu:** Methodology (equal); software (equal); writing – review and editing (equal). **Ying Chen:** Conceptualization (equal); funding acquisition (supporting). **Xuming Pan:** Conceptualization (lead); formal analysis (lead); funding acquisition (lead); methodology (lead); supervision (lead); writing – review and editing (equal).

## CONFLICT OF INTEREST

All authors declare that they have no conflicts of interest.

## Data Availability

The data presented in the study are deposited in the NCBI database (https://www.ncbi.nlm.nih.gov/) repository, accession numbers, lengths, and G&C contents are shown in Appendix Table A2.

## APPENDIX A

**TABLE A1:** Primers used in the polymerase chain reactions for ciliate SSU‐rRNA, ITS1‐5.8S‐ ITS2 rRNA, and mtSSUrRNA gene in the present study.

	Primer name	Primer sequence
SSU‐rRNA	Euk‐A	5′‐AAC CTG GTT GAT CCT GCC AGT‐3′ (Medlin et al., 1988)
	Euk‐B	5′‐TGA TCC TTC TGC AGG TTC ACC TAC‐3′ (Medlin et al., 1988)
ITS1‐5.8S‐ ITS2 rRNA	5.8S‐F	5′‐GTA GGT GAA CCT GCG GAA GGA TCA TTA‐3′ (Goggin, 1994)
	5.8S‐R	5′‐TAC TGA TAT GCT TAA GTT CAG CGG‐3′ (Goggin, 1994)
mtSSU‐rRNA	mt‐F	5′‐TGT GCC AGC AGC CGC GGT AA‐3′ (Hoek et al., 2000)
	mt‐R	5′‐CCC A(C)T ACC A(G)G TAC CTT GTG T‐3′ (Hoek et al., 2000)

**TABLE A2:** Newly sequenced genes in the present work

Taxon	Accession numbers	Lengths (bp)	G&C %	Sampling site
**Class Colpodea**				
*Colpoda inflata* pop1	MZ557833	1725	44.52%	Harbin
	MZ562697	1041	33.14%	
	MZ562700	721	38.00%	
*Colpoda inflata* pop2	MZ557838	1717	44.09%	Harbin
	MZ562692	1027	32.13%	
	MZ562693	656	38.87%	
*Colpoda* cf. *inflata*	MZ557804	1715	44.26%	Harbin
	MZ562691	1036	31.66%	
	MZ562698	722	37.67%	
*Colpoda grandis*	MZ562690	1711	45.30%	Harbin
	MZ562696	1066	32.55%	
	MZ562694	647	37.25%	
*Colpoda reniformis*	OL872369	1717	44.55%	Mudanjiang
	OL873584	1065	31.92%	
	OL873585	664	38.10%	
*Paracolpoda steinii* pop1	OL894854	1716	44.55%	Shuangyashan
	OL894851	1013	27.54%	
	OL894847	542	36.16%	
*Paracolpoda steinii* pop2	OL961825	1715	44.55%	Shuangyashan
	OL894855	1019	28.07%	
	OL894849	543	36.10%	
*Paracolpoda steinii* pop3	OL894848	1714	44.69%	Shuangyashan
	OL894858	1010	27.62%	
	OL894856	538	36.06%	
*Paracolpoda steinii* pop4	OL894852	1726	44.38%	Shuangyashan
	OL894853	1025	28.00%	
	OL894850	543	36.10%	
*Paracolpoda steinii* pop5	MZ562701	1714	44.34%	Shuangyashan
	MZ562699	1014	28.01%	
	MZ562695	533	36.40%	
